# YOC, A new strategy for pairwise alignment of collinear genomes

**DOI:** 10.1186/s12859-015-0530-3

**Published:** 2015-04-02

**Authors:** Raluca Uricaru, Célia Michotey, Hélène Chiapello, Eric Rivals

**Affiliations:** University of Bordeaux, CNRS / LaBRI, F-33405 Talence, France; University of Bordeaux, CBiB, F-33000 Bordeaux, France; MIG, UR 1077, INRA, 78026 Jouy-en-Josas cedex, France; MIA-T, UR 0875, INRA, BP 52627, 31326 Castanet-Tolosan cedex, France; LIRMM, UMR 5506, Computational Biology Institute, CNRS, University of Montpellier 2, Montpellier, France

**Keywords:** Comparative genomics, Whole genome alignment, Pairwise alignment, Anchor-based strategy, Collinear fragment chaining, Bacterial genomes

## Abstract

**Background:**

Comparing and aligning genomes is a key step in analyzing closely related genomes. Despite the development of many genome aligners in the last 15 years, the problem is not yet fully resolved, even when aligning closely related bacterial genomes of the same species. In addition, no procedures are available to assess the quality of genome alignments or to compare genome aligners.

**Results:**

We designed an original method for pairwise genome alignment, named YOC, which employs a highly sensitive similarity detection method together with a recent collinear chaining strategy that allows overlaps. YOC improves the reliability of collinear genome alignments, while preserving or even improving sensitivity. We also propose an original qualitative evaluation criterion for measuring the relevance of genome alignments. We used this criterion to compare and benchmark YOC with five recent genome aligners on large bacterial genome datasets, and showed it is suitable for identifying the specificities and the potential flaws of their underlying strategies.

**Conclusions:**

The YOC prototype is available at https://github.com/ruricaru/YOC. It has several advantages over existing genome aligners: (1) it is based on a simplified two phase alignment strategy, (2) it is easy to parameterize, (3) it produces reliable genome alignments, which are easier to analyze and to use.

**Electronic supplementary material:**

The online version of this article (doi:10.1186/s12859-015-0530-3) contains supplementary material, which is available to authorized users.

## Background

The huge number of genomes sequenced every day makes the development of effective comparison and alignment tools ever more urgent. Indeed, many microbiological applications rely directly on genome alignments, for instance micro-diversity and phylogenomic analysis of bacterial strains [1], assembly and annotation procedures for datasets of closely-related genomes [2] or prediction of maintenance motifs in non-model species [3]. Despite many efforts in this field and the availability of numerous genome aligners, some of which were specially designed for bacterial genomes (e.g., MGA [4], MAUVE [5], ProgressiveMAUVE [6], MUGSY [7], MAGIC [8]) and others that target more complex genomes (e.g., MUMmer [9], GRIMM-Synteny [10], CHAINNET [11], PipMaker [12]), none is yet completely satisfactory. Because genomes are subjected to a variety of complex mutational processes and rearrangements (substitutions, insertions/deletions, inversions, duplications, translocations, etc.), whole genome alignment (WGA) is a complex task that requires dedicated strategies.

**Figure 1:**
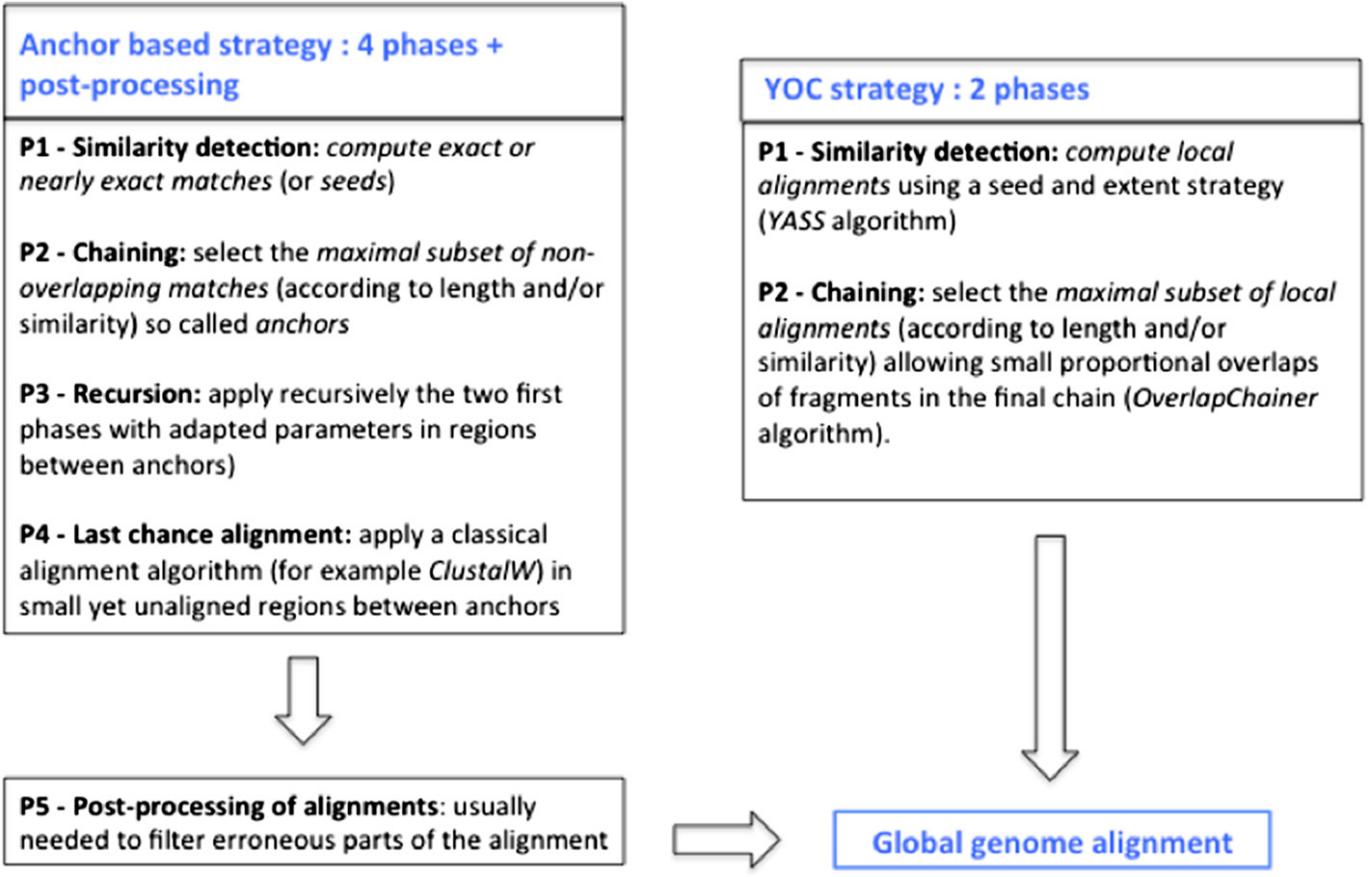
**Schematic description of two whole genome alignment strategies: the classical anchor strategy and the YOC strategy.** The anchor-based strategy includes 4 phases, which are usually followed by a post-processing step to filter the erroneous parts of the produced alignments (mainly related to the P4 “last chance alignment phase”). YOC method includes only two phases and does not require a post-processing step.

Classical WGA tools use a four phases, anchor-based strategy (see Figure 1) consisting of:

- Similarity detection (P1): computes pairs of genomic regions sharing sequence similarity, usually short, exact (or nearly exact) matches, e.g. MUMs, MEMs. These pairs of regions represent potential portions of the alignment.
- Chaining (P2): selects a maximal subset of non-overlapping matches (computed in P1) that form the backbone of the alignment, i.e. the anchors; the maximization criterion depends mostly on length and similarity. As the set of anchors can be ordered according to their genomic positions, it represents a chain: collinear if the relative order of the anchors is the same on both genomes and otherwise non-collinear.
- Recursion (P3): any two facing regions located between adjacent anchors on each genome are considered as smaller sequences and are aligned with the same procedure, i.e. by applying the first two phases (P1 + P2) recursively with adapted parameters, and complete the backbone with a second, complementary set of anchors.
- “Last chance alignment” (P4): uses classical alignment tools (e.g., ClustalW [13]) to compute global alignments between as yet unaligned facing regions. Alignments are performed and incorporated in the WGA based on different criteria depending on the aligner, for example, the difference in length between the two regions with MGA [4].

These four phases can be clearly identified in aligners targeting bacterial genomes, like MGA [4], which uses exact matches and a collinear chaining algorithm, LAGAN [14] (which is meant to deal with more divergent but still collinear sequences), which uses local alignments, collinear chaining and a dynamic programming alignment stage in the fourth phase, MAUVE [5] and ProgressiveMAUVE [6], which work with nearly exact matches and use a heuristic that produces a non-collinear chain. MUMmer (NUCmer) [9], which can deal with rearranged but slightly divergent genomes, implements a variation of this strategy, i.e. it uses exact matches that are clustered together in order to produce a non-collinear chain, but does not implement the fourth phase (‘last chance alignment’ phase). MAGIC is a highly sophisticated method that can be divided (in a very schematic manner) in two non-trivial phases, anchoring and non-collinear chaining, each of which is composed of numerous refinement stages [8]. Normally, MAGIC uses annotated genes as anchors, but can use any type of anchors as input. In the case of eukaryotic genomes, WGA tools freely adapt this strategy and generally use local alignments as anchors, which are ungapped (as in CHAINNET [11]) or gapped (for GRIMM-Synteny [10], PipMaker [12]), followed by clustering strategies, which are different from the chaining notion as they produce possibly overlapping clusters (and thus they do not give a true WGA). Given that here we specifically address whole genome alignment in bacteria, such methods are beyond the scope of the present paper.

The application of several whole genome aligners to large bacterial genome datasets [15,16] leads to three major observations:

- The tuning of parameters is a critical and complex step with many whole genome aligners. Two sets of parameters, even if they only differ slightly, can produce considerably different genome alignments. The choice of *ad-hoc* parameter settings is complicated and time consuming and depends on both the scientific question and the genomes under consideration (their number and sizes, their evolutionary distance, the presence or not of rearrangements).
- Most anchor-based methods suffer from flaws that lead to erroneous alignment of unrelated sequences. MAUVE alignment segment is an example of an alignment segment computed in the “last chance alignment” phase of MAUVE [5] for two *P. marinus* strains. In this alignment, regions with matching pairs of nucleotides are in the minority, thus it is clear that the two aligned sequences are unrelated. Such misalignments are possible for any aligner employing a “last chance alignment” phase if no proper inspection of the alignments is done in the end. Consequently, post-processing of the genome alignments is often required for these aligners.
- It is challenging and time-consuming to compare and evaluate the relevance of genome alignment results. This makes choosing the most appropriate tool for a given species or genome sample difficult.

### MAUVE alignment segment

The following segment was extracted from a MAUVE alignment of two P. marinus strains, with the start and end positions in the two sequences. The 137 length segment was included by MAUVE in the P4 “last chance alignment phase”, and it is is part of the final alignment, even though it is obvious that it aligns two unrelated sequences.

Considering these observations, in 2001, W. Miller [17] pointed out the development of dedicated methods to assess the quality of genome alignments as one of the crucial needs in comparative genomics. Thirteen years later this problem remains open and, given the recent efforts deployed for the Alignathon [18] competition, more popular than ever. Assessing the quality of a whole genome alignment is indeed a particularly difficult task, even in the simpler case of pairwise alignment. The first reason is that the real alignment is unknown and hence, exact measurement of its correctness is impossible. Secondly, alignment tools involve complex algorithms, which are often based on heuristic optimizations, and appropriate score functions are therefore needed to assess their quality. The third difficulty is the large quantity of data.

In recent years, the abovementioned issues were the subject of intensive studies and seve ral approaches have been proposed to bypass these limitations. Two different types of approaches are possible, see [19] for a comprehensive review. The first one consists in approximating the accuracy/correctness of the alignment. This kind of approach generally requires the use of external data such as gene annotation data [20,21] or simulated data [5,22]. The second approach consists in evaluating the reliability and/or the level of confidence of the resulting alignments. Such approaches are rooted in a wide range of technical foundations and include bootstrap-like strategies [23] or probabilistic models [24].

Aligning closely related bacterial genomes (for instance strains of the same species) should be one of the simplest cases for genome aligners, since the genomes are of moderate size (generally 1 to 6 Mb) and divergence times are short. Nevertheless, we observed that even in such cases, some WGA tools fail to capture more divergent regions, which are left out of the alignment, or conversely, tend to include wrong alignments of unrelated regions that need to be filtered out in a post-processing step [15,16]. With the aim of addressing this issue, we designed a more sensitive method for the similarity detection phase and a strategy to avoid the inclusion of badly aligned regions. We implemented this strategy in a new whole genome aligner named YOC, designed for robust pairwise alignment of collinear bacterial genomes. YOC provides several improvements: the strategy is simplified compared to other anchor-based tools and little parameter tuning is needed. Moreover, its sensitivity makes it possible to align more distantly related bacterial genomes. We also analyzed the quality and the reliability of the resulting alignments, which were extensively evaluated on several bacterial datasets. To this end, we introduce a quantitative criterion, GRA-FIL, based on the GRAPe software [25], and applied it to benchmark several tools. We show that this criterion measures efficiently the unreliable parts of the alignments, thus enabling rapid comparison of the performances of different genome aligners.

## Methods

### The YOC alignment method

Let us start with some considerations about the four phases, anchor-based strategy. First, “the last chance alignment” phase can obviously introduce unreliable alignment regions since it does not check whether the sequences it aligns are related. We propose to eliminate this phase. Second, the successive phases of similarity detection and chaining (P1, P2, P3) make parameter tuning difficult. However, these phases were justified by the use of short, exact (or nearly exact) matches as initial anchors, and are required to compensate for their lower capacity to capture more divergent regions. This choice also explains the low genome coverage of the resulting alignments on some very closely related but divergent genome pairs, like for instance in the endosymbiotic species *Buchnera aphidicola*.

To address this issue we propose to replace short matches (few dozen nucleotides) with local alignments (several hundred to several thousand nucleotides), as initial similarities. This choice has two advantages: it solves the observed lack of sensitivity and avoids the recursion phase, thereby considerably simplifying parameter tuning. For these reasons, our new strategy includes only two phases: similarity detection (P1) and chaining (P2) (see Figure 1).

### Phase 1: Similarity detection

The similarity detection phase (P1) is mainly responsible for the sensitivity of anchor-based methods, since the chaining phase only discards potential anchors. Therefore, the use of misfit similarity regions (short exact or nearly exact matches) explains the low coverage of the alignments even for related and similar pairs of genomes. Based on this observation, we propose to use spaced-seed local alignments in the first phase of the anchor-based strategy, as they are capable of detecting larger similarity regions that are more likely to make biological sense. We chose YASS [26], a seed-and-extend method, to generate these local alignments. Indeed, seed-and-extend methods are more suitable for divergent sequences, as they find significant similarity between sequences where short matches fail. YASS is a DNA pairwise local alignment tool based on an efficient and sensitive filtering algorithm that uses a flexible hit criterion to identify groups of seeds. Compared to the classical heuristic alignment tools (e.g., BLAST-like), which require an exactly matching k-mer, YASS uses the spaced seeds [27] technique, which increases sensitivity without losing specificity. The use of spaced seeds and local alignments (mostly BLAST-like) is not entirely new in the WGA field: e.g., MAUVE and ProgressiveMAUVE use inexact but ungapped matches as anchors, GRIMM-Synteny [10], PipMaker [12], LAGAN [14] and LASTZ [28], which use BLAST-like local alignments, while MAGIC [8] can be run with YASS local alignments.

A spaced seed is a pattern of #s and _s in which a # indicates an alignment position where a match is needed for the seed to have a hit, while a position with _ can be a match or a mismatch. An additional symbol @ can be used to denote matches or particular mismatches that correspond to transitions (purine to purine, or pyrimidine to pyrimidine). For instance #_#__# is a spaced seed of length 6 and weight 3, which will match an alignment window containing MdMddM where M denotes a match and d a difference. With this notation, a contiguous seed of length 6 has a pattern of ######. The main advantage of spaced vs contiguous seeds is the independence of their hits. Obviously, if a contiguous seed hits at say position i, it will very likely hit at position (i + 1), since the windows starting at these positions already share five of the six required matches. The pattern of a spaced seed forces the hits to be spread out along the alignment and thus be more independent of one another. Provided one looks for alignments longer than the seed length, the probability to get at least one hit is higher for a spaced than for a contiguous seed of equal weight [29]. This explains why spaced seeds improve sensitivity without losing specificity. This efficiency can be further enhanced by combining several spaced seeds, even if optimally spaced seeds are hard to design [30,31].

For YASS, a transition constrained seed model is used that capitalizes on the statistical properties of real genomic sequences. Comparative experiments have shown that, with the same degree of selectivity and a shorter running time, YASS is more sensitive than traditional approaches like Gapped-BLAST. Indeed, YASS detects similarities that cover about twice the overall length of those found by Gapped-BLAST, while keeping only local alignments with E-values below 10^−6^ [26]. For our similarity detection phase, YASS was set up with a commonly used pair of spaced seeds that were specifically optimized for the comparison of bacterial genomes: “#@_##_##_#__@_###, #_##@___##___#___#@#_#" (see reference [30] for more details on the design of sets of spaced seeds), and with the default E-value threshold of 10, which is intended to cope with divergence, regardless of how high it is.

### Phase 2: Chaining

Chaining algorithms seek to optimize several criteria, among which the total length of the chained fragments (i.e. similarities computed during the first phase: MEMs, MUMs, short local alignments, gene pairs, etc.), the distances between them, and the degree of rearrangement (for methods that deal with rearrangements) [5,6,32-34]. In the case of collinear chaining (neither translocations, nor inversions allowed), on which we focus in this paper, chaining methods generally maximize the total length of the chained fragments: given the set of n shared genomic intervals, i.e. fragments, the Maximum Weighted Chain (MWC) problem is solved in O (n log n) time by dynamic programming, when overlaps between adjacent fragments are forbidden [32,33].

In [35], we argued that the difficulty of using local alignments is that the chances that two adjacent fragments overlap are much higher than with short matches. At that point, we observed that such overlaps are commonly due to randomness, to methodological reasons during the fragment computation phase, or to biological phenomena, like tandem repeats. To avoid discarding relevant fragments in the chaining phase, it is useful to allow overlapping of adjacent fragments. Strategies for dealing with overlaps include accepting fixed, maximum length overlaps and trimming them (like in MAUVE and ProgressiveMAUVE) and segment match refinement (like in [36,37]). However, overlaps vary in size from extremely small to extremely large. Indeed, randomness and methodological problems are mostly responsible for short overlaps, while tandem repeats generate longer overlaps. Thus, accepting overlaps regardless of the fragment lengths is not the right solution. To get round this limitation, we extended the classical framework of the MWC in [35], by authorizing overlaps between fragments in the computed chain. We formalized the Maximum Weighted Chain with Proportional Length Overlap problem, where overlaps are proportional to the length of adjacent fragments. We also introduced the first algorithm to solve this problem (which takes quadratic time as a function of the number of fragments) and implemented it in a tool called OverlapChainer (OC). The algorithm is based on a box representation of a trapezoid graph [38], with an adaptation of the sweep line paradigm to this problem. In [35], the OC tool was tested on real data and compared to classical chainers with respect to simple quantitative measures, and its robustness was proved with respect to its only parameter, the overlap ratio (default value = 10%). In YOC, the tool presented here, we rely on OverlapChainer (OC) for the chaining phase. Our goal here is to prove the efficacy of this type of strategy when combined with spaced-seed local alignments in WGA, and to analyze the quality of the alignment results it produces.

To summarize, unlike classical WGA tools designed for similar genomes (like MGA, MUMmer (NUCmer), MAUVE, LAGAN, ProgressiveMAUVE), YOC focuses on almost collinear, highly divergent pairwise WGA, and simplifies the anchor based strategy by implementing only the first two phases (see Figure 1), without any refinement steps like realignment, filtering, or recursive alignment. Although a similar, simplified, two-phase strategy is already used in MUMmer [9], the solution is not entirely satisfactory. Its fragment computation phase is not appropriate for this simplified strategy because of its poor sensitivity (as it is based on exact matches).

**Figure 2:**
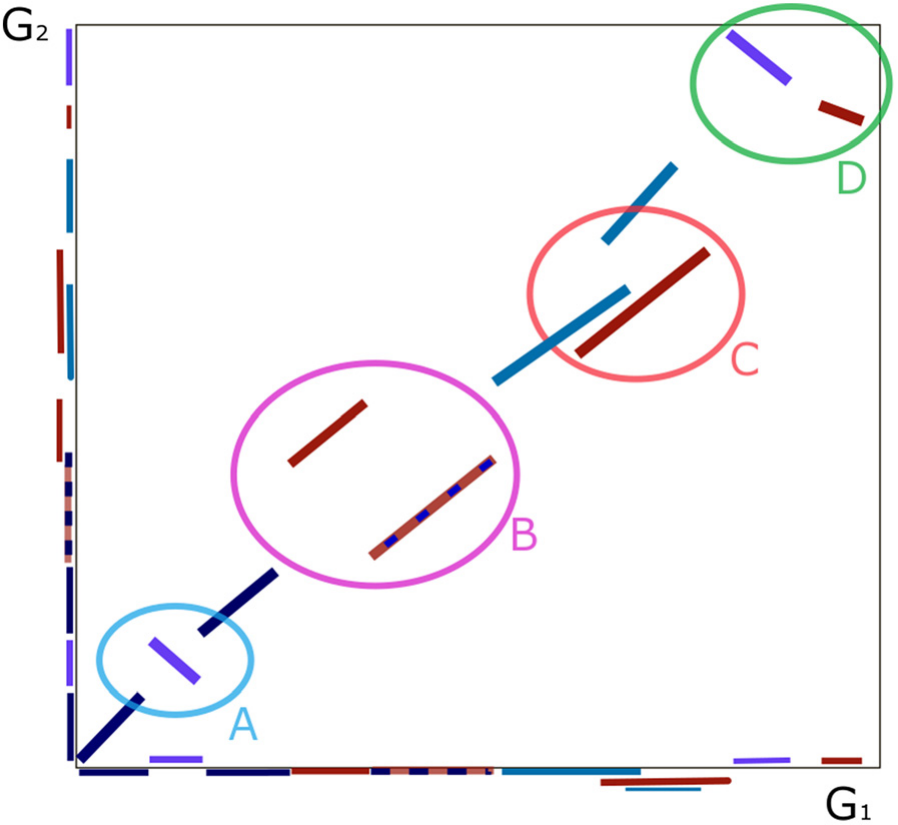
**A segment extracted from a MAUVE alignment of two** ***P. marinus*** **strains, with the start and end positions in the two sequences** ***.*** The 137 length segment was included by MAUVE in the P4 “last chance alignment phase”, when comparing two *P. marinus* strains. Therefore, the segment is part of the final alignment, even though it is obvious that it aligns two unrelated sequences.

The YOC strategy can be described as follows. Phase (1): YOC enhances the similarity detection phase by computing local alignments with YASS [26]; and phase (2): it chains the local alignments using a recent chaining algorithm, OverlapChainer (OC) [35]. As it relies on YASS, a pairwise local aligner, and OC, a collinear chaining method, YOC is designed for the alignment of collinear regions of genome pairs. However, it can be considered as an intermediate alignment strategy (between collinear aligners and aligners dealing with rearrangements), as it makes it possible to include locally inversed regions in the alignment (see Figure 2 for an example), meaning homologous DNA segments located on the forward strand in one genome, and on the reverse strand in the other. This is due to a straightforward transformation of YASS fragments before the chain is computed, namely switching the coordinates of the inversed fragments to make them collinear. Figure 2 shows the different cases our chaining procedure can handle and the ones it cannot.

**Figure 3:**
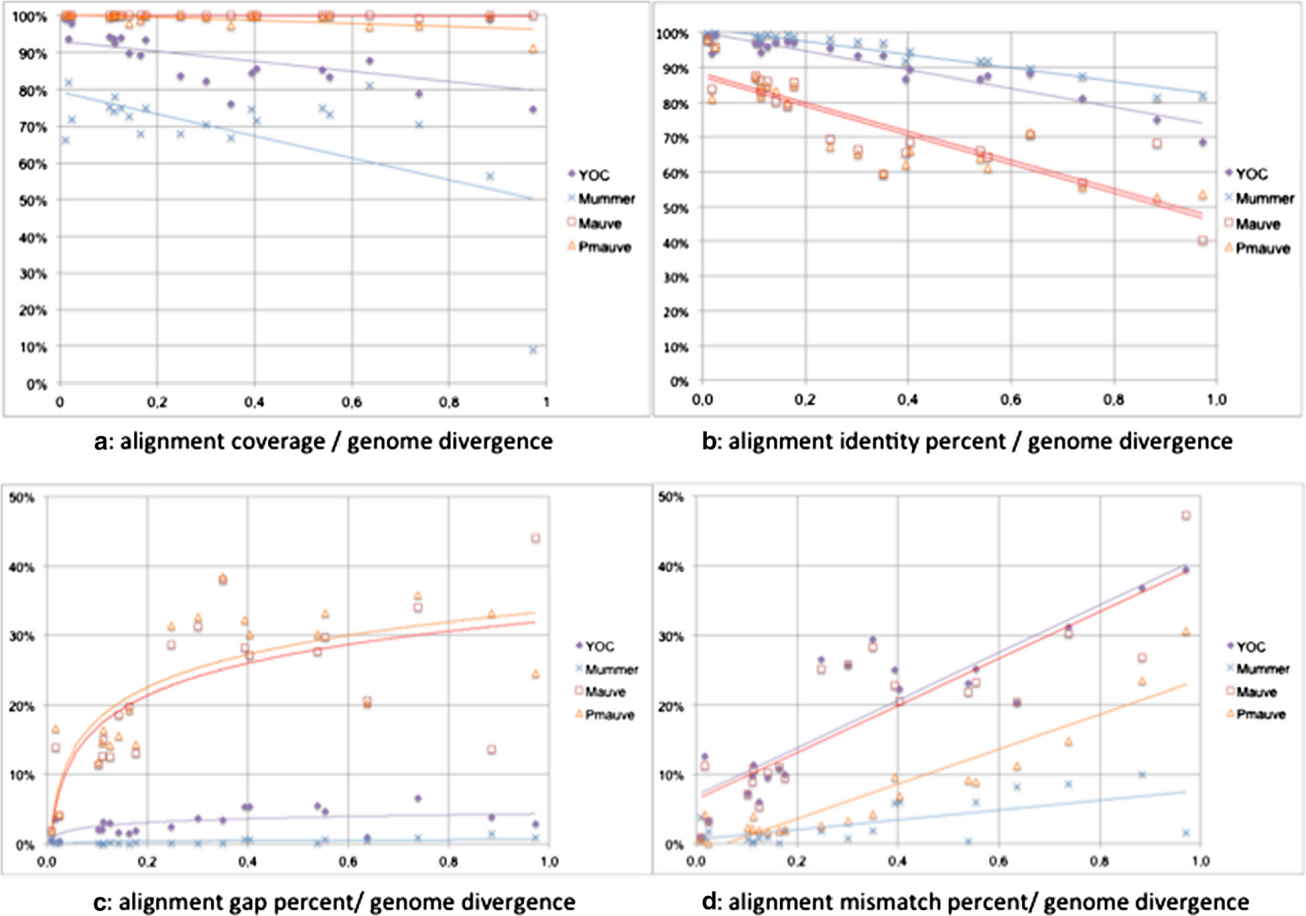
**Schematic representation of the OC chaining procedure, depicting the cases OC can deal with and the ones it cannot.** Overlap Chaining (OC) fragment chaining procedure illustrated schematically with a dotplot on an example composed of ten fragments (i.e., segments around the diagonal of the dotplot and their projections on the two axis), among which seven fragments were taken in the chain (i.e., dark blue, light blue, dotted blue and violet ones). The main chaining cases are summarized in the figure in four ellipses, namely: **(a)** a simple case represented by the two dark blue, collinear, non-overlapping fragments (classical chaining case) plus a local inversion (represented by the violet fragment), which becomes collinear after inversion with OC, and thus it is taken in the chain; **(b)** a translocation depicted by two red fragments: due to the collinearity constraints, only one fragment among the two can be taken in the chain, thus the algorithm prefers the “strongest” one, i.e. the dotted blue; **(c)** three overlapping fragments, among which only two are accepted in the chain (the light blue ones) as they overlap on less than 10% of their respective length; the red fragment is discarded as its overlaps are above the accepted threshold; **(d)** a double inversion represented by two inversed fragments from which only one can be taken in the chain (the violet one); by inversing the two fragments, OC generates a translocation-like situation, and thus it can only consider one fragment among the two.

### Genome datasets

#### Dataset 1 – 174 collinear pairs of bacterial genomes

We considered all collinear pairs of bacterial strains of the same species (based on the species name), with complete genomes like in release 5 of the MOSAIC database ([16], http://genome.jouy.inra.fr/mosaic). This dataset includes 174 pairs of genomes (see Additional file 1 for a complete list) that are considered to be collinear as (according to the criteria described in [16]), they do not include either inversions or translocations exceeding 20 kb in length.

#### Dataset 2–69 pairs of genomes in the Lactobacillus genus and in the Bacillus cereus species

We performed detailed analysis of genome alignments for 14 pairs of genomes of the *Lactobacillus* genus and 55 pairs of genomes of the *Bacillus cereus* species. These species were chosen because they mainly include collinear genomes (without rearrangements like inversions and translocations, according to the same criterion as in dataset 1) but in some cases, are nevertheless difficult to align due to high levels of divergence, even at the intra-species level.

Nineteen complete genomes of the *Lactobacillus* genus were extracted from Genome Reviews release 128 (2011), which included eight species with at least two complete genomes of two different strains. Fourteen intra-species pairwise genome alignments were produced and analyzed in detail in this study. See Additional file 2 for a complete list of these pairs of genomes.

*Bacillus cereus* is a gram-positive aerobic or facultative anaerobic spore-forming bacterium, part of the firmicutes group. Its chromosomes exhibit a high level of synteny and protein similarity with limited differences in gene content [39]. Eleven complete genomes of *B. cereus* were extracted from Genome Reviews release 128 (2011) and 55 pairwise genomes alignments were produced and analyzed in detail in this study. See Additional file 3 for a complete list of these pairs of genomes.

#### Dataset 3–21 collinear pairs exhibiting increasing genomic divergence

To examine the performance of WGA alignment tools with respect to the divergence rate, we selected 21 collinear pairs of genomes from the datasets used in a publication that introduced a measure of genome divergence called MUMi [40] (see Supplementary files 1 and 2 (http://jb.asm.org/content/191/1/91/suppl/DC1) of [40]). From the original datasets, only unique pairs without major rearrangements were used (pairs that do not include either inversions or translocations, according to the criteria described in [16]). Dataset 3 was composed of 21 genome pairs from 10 different bacterial species, exhibiting MUMi genomic distances ranging from 0.01 (very close pairs) to 0.97 (highly divergent pairs). See Additional file 4 for a complete list of these pairs.

#### Dataset 4 - Lactococcus lactis case study

*Lactococcus lactis* is a gram-positive bacterium extensively used in the production of buttermilk and cheese. It includes two sub-species: *L. lactis subsp. lactis* and *L. lactis subsp. cremoris*. As a case study, we analyzed the results obtained with several genome aligners on the pair composed of *L. lactis subsp. lactis*, IL1403 strain genome (AE005176_GR) and *L. lactis subsp. cremoris*, SK11 strain genome (CP000425_GR), which is also part of Dataset 3. To facilitate interpretation, we used the MOSAIC database to analyze and visualize the aligned regions [16] paying particular attention to their biological relevance.

### Benchmarking

In this section we detail the evaluation procedure used on the bacterial datasets presented above, with six genome aligners, including YOC. The resulting alignments were analyzed with respect to several qualitative and quantitative criteria described below.

### Genome aligners

Six genome aligners, all based on the anchoring strategy, were compared on the previously described genome datasets; two state-of-the-art aligners, MGA [4] and LAGAN [14], clearly target collinear genomes, three others, MAUVE [5], ProgressiveMAUVE [6] and MUMmer [9] are able to align either rearranged or collinear genomes whatever their type of rearrangements, while our new method, YOC, aligns pairs of collinear genomes (possibly including locally inversed segments but no translocated segments). To facilitate the comparison of the results, we used the XMFA alignment format produced by MAUVE and ProgressiveMAUVE aligners (for description, see http://darlinglab.org/mauve/user-guide/files.html) and wrote dedicated Perl scripts to transform the output of genome aligners that do not produce results in this format. Software versions and parameters are listed in Table 1. Despite numerous efforts, for practical reasons we were unable to include MAGIC in our benchmark, and thus we compared YOC to the published results of MAGIC on a dataset extracted from [8].

**Table 1:** **Genome aligner version and parameters**

	**Version**	**Program/Parameters**
MUMmer	3.22	’nucmer’ (parameters’–maxgap = 500 –coords’); delta-filter (options’-q -r -o 0’) and’show-aligns’.
MGA	mgadist-2003-03-18	’mkvtree’ (parameters’-dna -lcp -suf -tis -indexname’) and’mga.128seqs’ (parameters’-l 50 20 -gl 3000 -always –clustalw)’
LAGAN	1.1	with default parameters
MAUVE	2.3.1	with default parameters except for’–weight = 5000’ and’–output-alignment’ for XMFA file output
ProgressiveMAUVE	2.3.1	with default parameters except for’–output-alignment’ option for XMFA file output
YOC	1.0	with default parameters : for ‘YASS’ (parameter ‘E-value threshold’: 10) and for Overlap Chainer (parameter ‘overlap ratio’ : 10%).

### Quality criteria of genome alignments

Several quantitative and qualitative criteria were used to compare genome alignments produced by different tools (classically used in genome alignment):

- the number of aligned segments, which represents a measure of the fragmentation of the genome alignment,
- the length of the alignment expressed as the number of aligned positions,
- the number of identical residues in the alignment, which is the only value that is easy to compare and analyze between aligners,
- the mean coverage of the alignment, a classical criterion defined as the mean proportion of non-gap characters aligned in each genome, i.e. mean between the matches + mismatches in the aligned regions of genome 1 and 2, divided by the size of the genome 1, respectively 2,
- the percentage of identities in the alignment, defined as the number of aligned identical residues in the alignment divided by the length of the alignment,
- the percentage of gaps in the alignment defined as the number of gap positions in the alignment divided by the length of the alignment,
- the percentage of mismatches in the alignment defined as the number of aligned non identical residues in the alignment divided by the length of the alignment.

An original quality criterion, named GRA-FIL, was defined based on a filtration procedure consisting in post-processing raw alignments with the GRAPe [25] software. GRAPe is a probabilistic genome aligner capable of quantifying the uncertainty of each position of the alignment with a posterior probability. GRAPe was applied on each pairwise genome alignment obtained by each aligner with the aim of filtering the parts of the alignments that are suspected to be spurious and incorrectly aligned. In order to cope with the lengths of the sequences (as GRAPe is too slow to be systematically applied at large scale), we partitioned the alignments in adjacent, 500 position length blocks, and used GRAPe to realign every such short region. The procedure consists in eliminating (filtering) blocks that have at least half of their positions with a posterior probability of being incorrectly aligned greater than 0.95 (i.e. regions that are predicted by GRAPe to be *unalignable* or to be part of insertions and gaps). Using this procedure, for each alignment, we computed the length of the regions filtered with GRAPe (as the number of aligned positions or as the percentage of the alignment length), a criterion we named GRA-FIL, which is a precise indicator of the proportion of low-quality regions in a genome alignment. The GRA-FIL procedure is very similar to the one used in the Alignathon competition, which is based on another probabilistic aligner, PSAR [41].

Finally, we defined a criterion of biological relevance based on the analysis of orthologous gene positions in the aligned regions. The orthologous genes were extracted from the OMA database [42]. We measured the number of known orthologous genes entirely included in the same aligned segment, the number of orthologous genes entirely included in unaligned regions, and the number of these genes that overlap the two types of segments. The underlying assumption is that the most accurate and biologically relevant alignment is the one including a maximum number of orthologous genes (assumed to be vertically inherited) in the same aligned segments.

## Results

Below we summarize and discuss the results we obtained with our two-phase anchor based strategy, YOC (described in Section “The YOC alignment method” and Figure 1), compared to five classical anchor based tools on the three datasets described above. The comparisons were conducted based on the criteria defined in Section “Quality criteria of genome alignments”. To this we add a comparison of YOC results with MAGIC results on a dataset extracted from [8] (see subsection “Magic dataset case study”).

On Dataset 1, we observed high variability of the overall quantitative results obtained with the different tools, e.g. the difference between the mean coverage obtained with MGA and that obtained with MAUVE ranged from −24% to 2% (meaning that there is at least one pair of genomes for which MGA’s mean coverage is 24% below that of MAUVE, and at least one pair for which MGA’s coverage exceeds that of MAUVE by 2%).

Given that similar tools yield such different outputs, results cannot be directly used, and judging the best alignment tool for a given pair becomes extremely difficult. Indeed, the results depend to a great extent on the profile of the genomes: their divergence rate, as well as whether or not they are collinear. Moreover, quantitative results alone are not enough to judge the quality of an alignment. To address this question, we further examined the quality of the alignments using the GRA-FIL criterion described in the previous section.

### Global quality of genome aligners (Dataset 1)

The results obtained with four tools: MGA, LAGAN, MAUVE and YOC, in terms of mean coverage before and after filtering with the GRA-FIL criterion are listed in Table 2 for the 174 pairs of genomes in Dataset 1 (see Additional file 1 for more details). As can be seen in Table 2, the mean coverages ranged from 74% to 100% for MGA, MAUVE and YOC, while LAGAN achieved 100% mean coverage each time. However, when we look at the mean coverages after filtering with the GRA-FIL criterion, we observe that, taking MAUVE for example, in some cases coverage drops to 58%. Indeed, if we analyze the mean coverage that was lost by filtering, we deduce that MGA, LAGAN and MAUVE can produce important quantities of spurious alignments: up to 13% of the alignments were filtered for MGA, respectively 21% for LAGAN and 35% for MAUVE. LAGAN’s results can be explained by the fact that it leaves no regions unaligned, regardless of their similarity (in the final phase, it aligns every remaining pair of unaligned regions with a dynamic programing alignment procedure). MAUVE also forces the alignment of unrelated sequences and its authors are aware of the problem, which is discussed in [20], and was claimed to be solved in ProgressiveMAUVE [6] (which includes a post-alignment filtration step). Finally, YOC clearly exhibits the lowest levels of alignment filtered by GRAPe (with up to 4% filtered and 0.8% on average). To conclude, all four phases, anchor-based tools that do not post-filter their alignments include incorrectly aligned regions in their output. In contrast, with only two phases, YOC considerably reduces the need for filtration.

**Table 2:** **Evaluation of the quality of the alignment results produced by four genome aligners on 174 collinear pairs of bacterial genomes**

	**Raw coverage (%)**			**Coverage after GRAPe filtering (%)**			**GRA-FIL criterion (%)**		
	**Average**	**Min**	**Max**	**Average**	**Min**	**Max**	**Average**	**Min**	**Max**
**MGA**	91.5%	74%	99%	89%	70%	99%	2.5%	0%	13%
**LAGAN**	100%	100%	100%	95%	79%	100%	5%	0%	21%
**MAUVE**	95.2%	78%	100%	92.8%	58%	99%	2.4%	0%	35%
**YOC**	93.3%	74%	100%	92.5%	72%	100%	0.8%	0%	4%

Values correspond to the average, the minimum and the maximum alignment coverages before and after applying the GRA-FIL filtering procedure, as well as the average, the minimum and the maximum for the GRA-FIL criterion (i.e., the percentage of likely erroneous alignment positions). Full results are given in Additional file 1.

### Assessment of the reliability of intra-species pairwise genome alignments (Dataset 2)

Based on the preliminary results listed in Table 2, we decided to compare five genome aligners, MGA, MUMmer (NUCmer), MAUVE, ProgressiveMAUVE and YOC, using the intra-species pairs of genomes from Dataset 2. Given that MUMmer (NUCmer) uses exact matches (unique or not) and that it does not include the ‘last chance alignment phase’, the length of its alignment can serve as a lower bound of the number of alignable positions. Clearly, the GRAPe filtration should only affect its results marginally. Two sub-datasets of the Dataset 2 were extensively analyzed, the results are listed in Tables 3 and 4 (see Additional files 2 and 3 for more details). Table 3 presents the results regarding the alignment quality before and after filtration of alignments with the GRAPe procedure. These results indicate that the genome aligners can be grouped in three categories:

1. MUMmer (NUCmer) produced almost perfect alignments (99.9% of identity for *Lactobacillus*, 92.3% for *Bacillus cereus*) of limited length: on average 2.0 Mb for *Lactobacillus* (mean coverage: 76.6%) and 3.6 Mb for *Bacillus cereus* (mean coverage: 69.4%). NUCMER alignments are split into a large number of aligned segments (108 aligned segments for *Lactobacillus* and 621 for *Bacillus cereus*). As expected, the filtration procedure has almost no effect on NUCMER alignments.
2. MAUVE and ProgressiveMAUVE yielded the longest alignments (on average 2.9 million positions for *Lactobacillus* and 6.0 million positions for *Bacillus cereus,* i.e. 100% coverage) including only a few long segments (respectively 2/1 on average with MAUVE/ProgressiveMAUVE in *Lactobacillus*, and 3/33 with MAUVE/ProgressiveMAUVE in *Bacillus cereus*). Very long segments suggest that large genomic regions are orthologous and well conserved. However, we observed that: (i) the percentage identity of the alignments was quite low especially in *B. cereus* (mean: 65%), (ii) the filtration by GRAPe considerably shortens their alignment and splits them into numerous segments. Indeed, after filtration, their mean alignment lengths dropped to 2.4 million positions for *Lactobacillus* (mean coverage: 91%), and to 4.4 million positions (mean coverage 83% with MAUVE) or 4.2 million positions (mean coverage 81% with ProgressiveMAUVE) for *Bacillus cereus*.
3. MGA and YOC behaved differently: filtration had a moderate effect in terms of alignment length or number of alignment segments. The original alignment lengths of 2.3 or 2.4 (MGA and YOC respectively) were reduced to 2.2 million positions for *Lactobacillus* (around 82% of mean coverage with MGA and 89% of mean coverage with YOC). The results for the *Bacillus cereus* group were similar with the two aligners, with a length after filtration of around 4.2 million positions (around 80 and 81% of mean coverage with MGA and YOC) and a high percentage identity (around 90% on average). Note that the number of identities with all the aligners remained almost the same after filtration, suggesting that solid regions of the alignment are kept and that removed regions had much lower levels of identities. Moreover, after filtration, the alignment lengths obtained with MAUVE and ProgressiveMAUVE were equal to those produced with YOC.

**Table 3:** **Quality of raw and filtered genome alignments produced by five genome aligners according to classical quality measures**

***Lactobacillus*** **14 intra-species alignments**			
	**Mean number of segments before filtering**	**Mean alignment length [Cov] before filtering**	**Mean number of identities [%id] before filtering**
**NUCMER**	108	2 010 305 [76.6]	1 985 563 [99.9]
**MGA**	33	2 267 771 [83]	2 155 141 [95.2]
**MAUVE**	2	2 895 734 [100]	2 389 905 [83.7]
**PMAUVE**	3	2 898 388 [99.7]	2 376 004 [83.1]
**YOC**	70	2 427 113 [90.8]	2 338 377 [96.2]
	**Mean number of segments after filtering**	**Mean alignment length [Cov] after filtering**	**Mean number of identities [%id] after filtering**
**NUCMER**	108	2 010 302 [76.6]	1 985 561 [99.9]
**MGA**	91	2 190 409 [81.6]	2 146 901 [98.0]
**MAUVE**	134	2 446 079 [91.7]	2 370 060 [96.8]
**PMAUVE**	111	2 419 589 [91]	2 365 075 [97.7]
**YOC**	109	2 368 669 [88.7]	2 328 734 [98.2]
***Bacillus cereus*** **55 intra-species alignments**			
	**Mean number of segments before filtering**	**Mean alignment length [Cov] before filtering**	**Mean number of identities [%id] before filtering**
**NUCMER**	621	3 624 990 [69.4]	3 371 181 [92.3]
**MGA**	132	4 544 305 [83.5]	3 827 363 [83.4]
**MAUVE**	1	6 082 756 [100]	3 963 239 [65.5]
**PMAUVE**	33	6 043 087 [100]	3 869 392 [64.3]
**YOC**	313	4 448 646 [83.4]	3 907 562 [87.1]
	**Mean number of segments after filtering**	**Mean alignment length [Cov] after filtering**	**Mean number of identities [%id] after filtering**
**NUCMER**	621	3 624 978 [69.4]	3 371 172 [92.3]
**MGA**	418	4 186 007 [79.8]	3 790 894 [89.7]
**MAUVE**	564	4 418 643 [83.3]	3 884 906 [87.0]
**PMAUVE**	522	4 269 387 [81.2]	3 824 790 [88.7]
**YOC**	470	4 266 745 [81.5]	3 887 006 [90.3]

Part 1 of the table corresponds to the 14 intra-species pairwise genome alignments of the *Lactobacillus* genus. Part 2 of the table corresponds to the 55 intra-species pairwise genome alignments of the *Bacillus cereus* species. The software compared are: MUMmer (NUCmer), MGA, MAUVE, ProgressiveMAUVE (PMAUVE) and YOC. Values correspond to classical quality criteria (mean number of segments, mean alignment length, mean alignment coverage [Cov], mean number of identities and mean percentage of identities [%id]) before and after the GRAPe filtration procedure described in Section “Quality criteria”. Full results are given in Additional files 2 and 3.

**Table 4:** **Quality of genome alignments produced by five genome aligners according to our new qualitative criterion**

	**GRA-FIL average (in number of pos. and [%])**	**GRA-FIL minimum (in number of pos.)**	**GRA-FIL maximum (in number of pos.)**
***Lactobacillus*** **14 intra-species alignments**			
NUCMER	2 [0.00%]	0	5
MGA	77 362 [1.47%]	59	157 619
MAUVE	449 655 [8.31%]	0	802 632
PMAUVE	478 799 [8.66%]	0	866 011
YOC	58 445 [1.27%]	0	125 821
***Bacillus cereus*** **55 intra-species alignments**			
NUCMER	10 [0.00%]	0	158
MGA	358 298 [7.63%]	143 412	436 054
MAUVE	1 664 114 [22.45%]	592 079	2 950 027
PMAUVE	1 773 700 [24.70%]	666 595	3 390 532
YOC	181 900 [4.5%]	71 851	262 813

Part 1 of the table corresponds to the 14 intra-species pairwise genome alignments of the *Lactobacillus* genus. Part 2 of the table corresponds to the 55 intra-species pairwise genome alignments of the *Bacillus cereus* species. The software compared are: MUMmer (NUCmer), MGA, MAUVE, ProgressiveMAUVE (PMAUVE) and YOC. The values in the table correspond to the average, the maximum and the minimum results using the GRA-FIL criterion, i.e. the number and the percentage of the likely erroneous alignment positions that were filtered by the GRAPe filtration procedure described in Section “Quality criteria”. Full results are given in Additional files 2 and 3.

Table 4 summarizes the amount of alignment filtered by GRAPe with each aligner and all genome pairs of both datasets and confirms these results. It turns out that the average amount of positions filtered by the GRAPe procedure (GRA-FIL) is very high in both MAUVE (449.655 positions = 8.31% for *Lactobacillus* and 1.664.114 positions = 22.45% for *Bacillus cereus*) and ProgressiveMAUVE (478.799 positions = 8.66% for *Lactobacillus* and 1.773.700 positions = 24.70% for *Bacillus cereus*), compared with MGA (1.47% and 7.63% for *Lactobacillus* and *Bacillus cereus* respectively) and YOC (1.27% and 4.50% for *Lactobacillus* and *Bacillus cereus* respectively). With *Bacillus cereus,* an average of 22%, resp. 25%, of the MAUVE/ProgressiveMAUVE alignments were considered unreliable and removed by GRA-FIL, which filtered only 4.5% of YOC alignments. Surprisingly, the filtration ratio for ProgressiveMAUVE was high despite the fact that ProgressiveMAUVE already includes a quality filtering step.

To summarize, based on the GRA-FIL quality criterion, the results in Tables 3 and 4 suggest that MAUVE and ProgressiveMAUVE extend their alignments by including regions of questionable similarity, while in only two phases, YOC produces the most reliable alignments of all. Moreover, according to its coverage of alignments and the number of identities, YOC directly outputs alignments similar to those obtained with MAUVE and ProgressiveMAUVE after filtration with GRAPe. It is also interesting to note an unexpected result: ProgressiveMAUVE does not systematically produce better results than MAUVE. This may be due to the fact that ProgressiveMAUVE was designed and tuned for the alignment of multiple genomes.

### Aligner performances with respect to the genome divergence (Dataset 3)

The way of life of a bacterium may affect the rapidity at which its genome diverges within a species. This raises an important question: how does the divergence level of a genome pair impact the performance of WGA? Using a dataset for which the divergence level was previously measured at the intra-species level [40], we applied four WGA tools and compared them using four criteria. Results are shown in Figure 3 (see Additional file 4 for more details): coverage (Figure 3a), percentage of identities (Figure 3b), percentage of gaps (Figure 3c), and percentage of mismatches (Figure 3d) of the alignments. Results in Figure 3a indicate that increasing levels of divergence have little effect on alignment coverage. MAUVE and ProgressiveMAUVE had the highest coverage values, close to 100%, regardless of the genome divergence levels. A slightly linear decrease of MUMER/YOC coverages was observed with an increase in the level of divergence. YOC coverages were between those of MAUVE/ProgressiveMAUVE and MUMmer. Figure 3b shows that increasing the level of divergence mainly affected the alignment mean percentage of identity for all four tools (which decreases linearly), but had more drastic effects on MAUVE and ProgressiveMAUVE alignments than on YOC and MUMmer alignments. To better understand the origin of this result, we computed the percentage of gaps (Figure 3c) and the percentage of mismatches (Figure 3d) of the aligned regions. Results indicate that with all four tools, the number of mismatches increases with the divergence rate (Figure 3d). But more surprisingly, MAUVE and ProgressiveMAUVE alignments included high rates of gap positions, which reached 40% of the alignment, even for moderate levels of divergence (divergence > 0.2, according to MUMi values). This phenomenom was not observed in either MUMmer, or in YOC alignments, for which the proportion of gaps remained low, regardless of the divergence rate. To summarize, taken together, these results indicate that YOC offers a good compromise between coverage and the percentage of identity, at any divergence rate.

**Figure 4:**
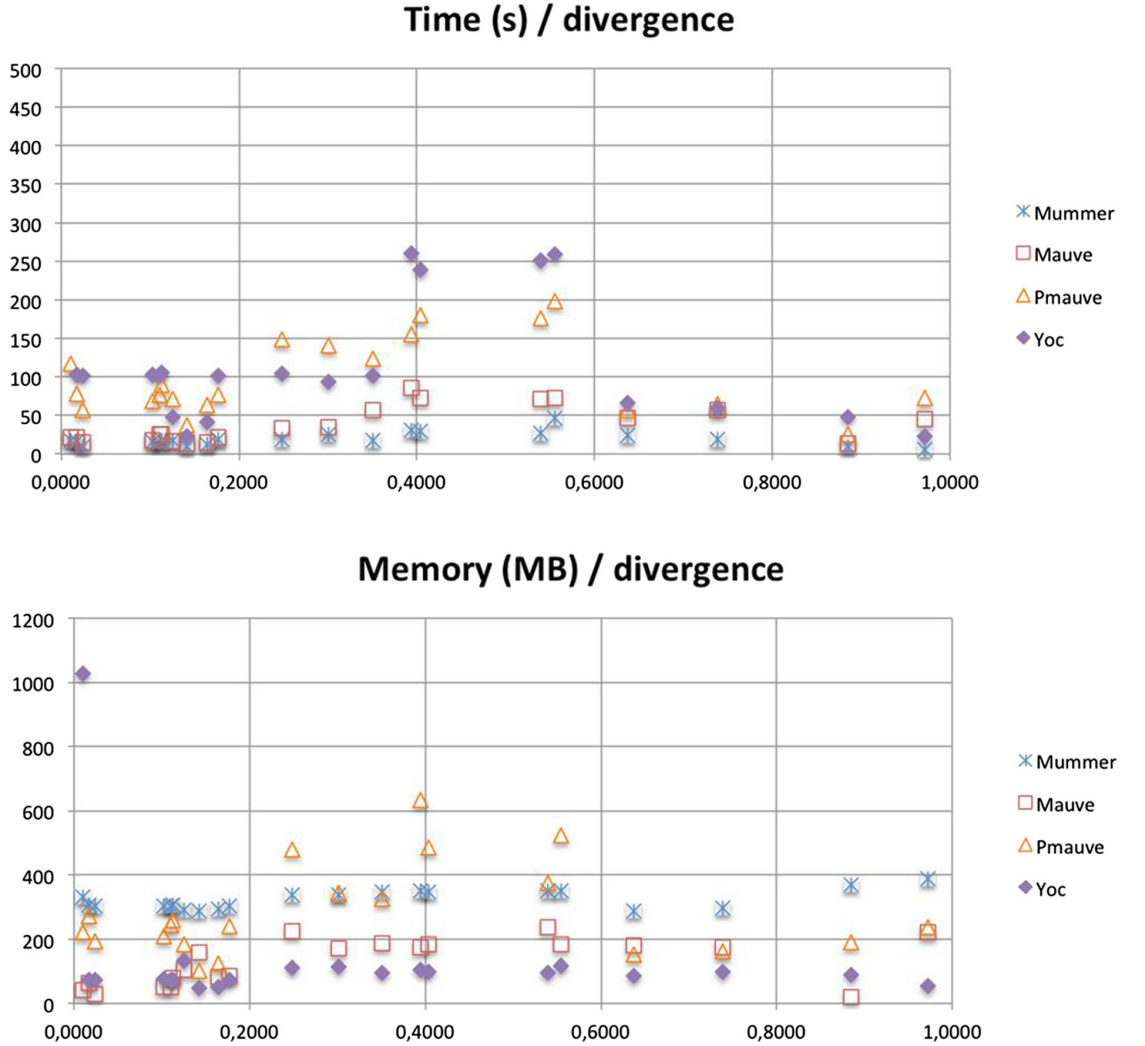
**Comparison of MUMmer (NUCmer), MAUVE, ProgressiveMAUVE and YOC alignments on 21 pairs of genomes of increasing divergence.** Plots represent alignment mean coverage **(a)**, percentage of identity **(b)**, percentage of gaps **(c)**, and percentage of mismatches **(d)**, with respect to genome divergences measured with MUMi values (X-axis). Linear (**a**, **b**, **d**) and logarithmic (**c**) regression curves are plotted together with raw values for the 21 aligned genome pairs. Full results are given in Additional file 4.

Finally, Figure 4, shows the results of the analysis of the effects of divergence on the computational ressources (time and memory) for each of the four tools. As one can see in Figure 4, there does not appear to be any correlation between divergence and the use of computational resources in any of the tools. While YOC’s needs in terms of time may exceed those of the other approaches, in terms of memory its needs are generally lower. The high running times of YOC are explained by the way YASS is used, namely with an E-value of 10, in order to avoid any filtering before the chaining phase and to make it possible to detect similarity even in extremely divergent regions. Due to this tuning, YASS can produce up to several hundred thousand fragments (especially in close genomes with numerous repeated regions), thus the time needed to process these fragments may be high but remains within reasonable limits.

**Figure 5:**
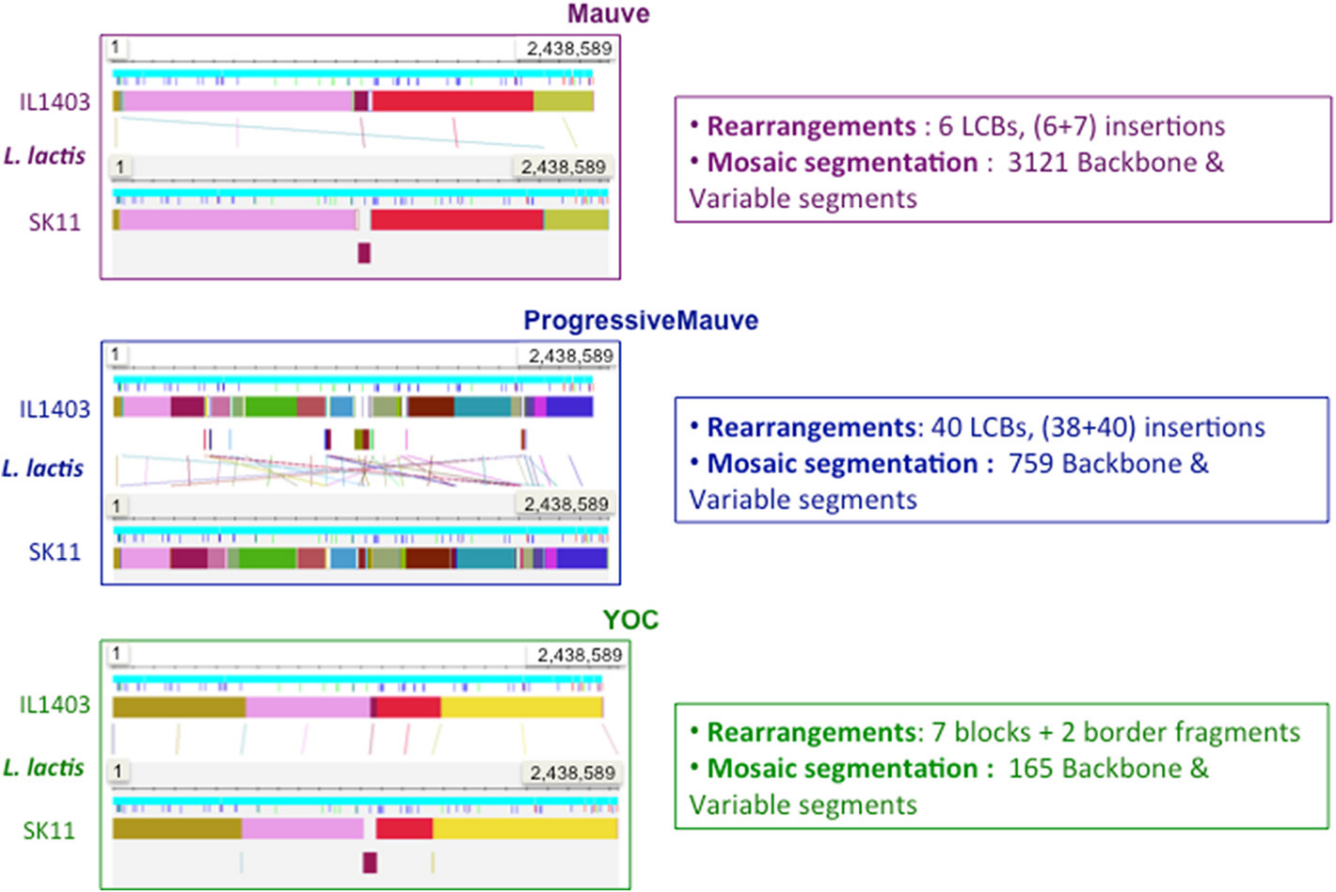
**Comparative time and memory performances on Dataset 3, with respect to increasing divergence levels.** Compared to MUMmer (NUCmer), MAUVE and ProgressiveMAUVE, on Dataset 3, YOC presents higher running times but lower memory needs (except for one case), regardless of the divergence level (computed with MUMi distance measure). Indeed the divergence level does not seem to affect the computational measures of any of the four tools.

### *Lactococcus lactis* case study (Dataset 4)

Table 5 and Figure 5 show the results of a detailed analysis of three genome alignments produced by MAUVE, ProgressiveMAUVE and YOC on two *Lactococcus lactis* genomes, which were post-processed by filtering the low quality alignment regions (with more than 20 consecutive gaps) and the weakly conserved regions (with less than 76% of identity). The filtered alignments, i.e. backbone alignments, for each of the three tools were incorporated in the Mosaic database [16] to facilitate the comparison. When analyzing the genome alignments we refer to two types of regions that partition the compared bacterial genomes: backbone segments (i.e. regions conserved in both genomes) and variable segments (i.e. regions that are either specific to or variable in one of the aligned genomes) [16]. The backbone alignment is composed of all the aligned backbone segments taken together.

**Table 5:** ***Lactococcus lactis*** **case study**

	**MAUVE**	**ProgressiveMAUVE**	**YOC**
***Lactococcus lactis IL1403 compared to SK11***			
#Backbone segments	3121	759	165
Mean coverage	64%	77%	79%
Backbone identity%	89%	86%	85%
#included orthologs	370 [27%]	1 173 [85%]	1 287 [92%]
#overlapping orthologs	957 [69%]	193 [14%]	33 [2%]
#excluded orthologs	68	29	75
Orthologs coverage	82.9%	97.4%	95.2%

Basic indicators and biological relevance of three genome alignments (post-processed into backbone segments) produced by MAUVE, ProgressiveMAUVE and YOC for the comparison of two *Lactococcus lactis* genomes: the *subsp. lactis*. IL1403 strain genome and the subsp. *cremoris*. strain SK11 genome. The criteria are the number of backbone segments (#Backbone segments), the mean coverage of the backbone (Mean coverage), the percentage of identity of the backbone (Backbone identity%), the number of orthologs included in the backbone (#included orthologs), overlapping the backbone (#overlapping orthologs) and excluded from the backbone (#excluded orthologs), and the orthologous cumulative length coverage in the backbone (Orthologs coverage) The orthologous genes were extracted from the OMA database [42].

The measures of the reliability of the backbone alignments obtained using the basic indicators (number of segments, mean coverage and percentage of identity) differed considerably between the three aligners: MAUVE tended to produce a highly fragmented (3121 segments) and low-coverage (64% mean coverage) but a highly conserved backbone (89% identity), the results of ProgressiveMAUVE were intermediate, with a less fragmented backbone (759 segments), medium coverage (77%) and good percentage of identity (86%). YOC produced the best results, with few segments (165), high coverage (79%) and a good percentage of identity (85%).

The biological relevance of the three alignment backbones was evaluated by analyzing the position of the orthologous genes in the backbone segments. The results in Table 5 indicate that 92% of the orthologous genes are correctly included in the YOC backbone, compared to only 27% in the MAUVE backbone. Indeed, in MAUVE, 68% of the orthologs are split between aligned and unaligned regions (i.e., backbone and variable segments). ProgressiveMAUVE produced quite good results, with 85% of the orthologous genes completely and correctly included in the backbone segments. Even though in terms of the total number of orthologous positions included in the alignment backbone (this means taking into account orthologs that overlap both backbone and variable segments) ProgressiveMAUVE obtained better scores than YOC (97.4% compared to 95.2%), the corresponding ProgressiveMAUVE backbone segments tended to hatch the orthologous genes and were less relevant from a biological viewpoint. This phenomenon is clearly illustrated in Figure 5, which shows the backbones of MAUVE, ProgressiveMAUVE, and YOC. The backbones of the first two are split in smaller segments than that of YOC. Indeed, most orthologs do not fit in one segment in MAUVE and ProgressiveMAUVE alignments, while they do in those of YOC.

### MAGIC dataset case study

As we were unable to run MAGIC, we applied YOC on a bacterial set used to assess MAGIC’s performance in [8] (i.e., the 12 pairs of genomes listed in Tables three and six of MAGIC paper [8]). MAGIC’s raw results on this dataset were extracted as such from [8] and correspond to the number and the coverage of Reordered Free (RF) segments obtained from curated pairs of orthologs refined in a multi-step pre-processing phase and iteratively post-processed in a clustering phase. We compared these values to YOC raw fragment number and coverage. Our results showed lower performances by YOC for 11 out of the 12 pairs (YOC coverage: 15 to 84%, MAGIC/RF coverage: 34 to 99%). But interestingly, for one of the 12 pairs (*Buchnera aphidicola*), YOC’s performance was better according to the alignment coverage (YOC: 98-99%, MAGIC/RF: 93%). Even though the results are not entirely comparable between the two tools and the dataset clearly does not fit YOC’s application area (11 among the 12 pairs are highly rearranged), they confirm YOC’s ability to align highly divergent genomes. Indeed, MAGIC is a versatile and sophisticated tool that, unlike YOC, appears to be perfectly adapted to dealing with rearrangements (as we observed in 11 out of the 12 pairs). Nonetheless, on the *Buchnera aphidicola* pair, which is highly divergent but rearrangement free, YOC showed a clear advantage over MAGIC with respect to coverage. For complete results see the Additional file 5.

## Discussion

In this paper, we present a new tool for pairwise alignment of collinear genomes, called YOC, which includes only two phases of the classical four phase anchor-based strategy: the first for detecting local alignments as potential anchors and the second to chain the similarities that will form the alignment. This simplified algorithm leaves out recursion and avoids the "last chance alignment” phase.

We compared and benchmarked YOC with several well-known whole genome aligners on *a priori* easy cases: pairwise alignments of bacterial genomes of the same species. To evaluate the impact of the “last chance alignment” phase, we use GRAPe to filter out unreliable parts of the alignments on several datasets. We observed that MGA, MAUVE and ProgressiveMAUVE, which all include the third and fourth phases of the anchor-based strategy, yielded alignments with high genome coverage, but of which a considerable proportion was detected as being unreliable. On average, of all *B. cereus* pairs, 20% of ProgressiveMAUVE’s alignment was filtered out. After filtration of these regions, the percentages of identity of the original and final alignments were almost the same, strongly suggesting that regions filtered by GRAPe are of poor quality and should be removed. It also turns out that after filtration, these alignments exhibited the same coverages as those output by YOC. In contrast, alignments computed with YOC were much less altered by filtration, e.g. only 4.5% on average over all *B. cereus* cases. This conclusion was corroborated on Dataset 3, which revealed MAUVE’s and ProgressiveMAUVE’s tendency to include an increasing number of mismatches and gaps for higher divergence levels, compared to YOC, which offers a good compromise between coverage and percentage of identities.

This is in favor of the simpler, two phases, strategy implemented in YOC. Recursion is avoided by the use of more sensitive local alignments. YOC does less work but achieves similar levels of coverage and identity to a sophisticated aligner like ProgressiveMAUVE. Moreover, it captures the pairs of regions that can be reliably aligned. This was confirmed by looking at the positions of orthologous genes in the alignment backbones of *L. lactis* genomes. YOC alignments were those that included the largest numbers of complete orthologs in the aligned regions. Finally, its alignments comprised fewer segments than those of MGA, MAUVE, or ProgressiveMAUVE.

YOC uses YASS, a highly sensitive local alignment software in phase one, and OverlapChainer, a chaining algorithm allowing for proportional overlaps between the anchors in phase two. Both procedures are relatively fast, use little memory and have few parameters to tune. Although it is not the ultimate genome aligner, we argue that in practice, YOC combines important advantages:

1. Simplicity of the algorithm: only similarity detection and chaining are performed, which avoids including badly aligned regions.
2. Simplicity of use: as the spaced seeds are already optimized for bacterial genomes, YOC only requires the tuning of two parameters: (i) the E-value threshold for YASS, the higher the better if the goal is to ensure high sensitivity regardless of the level of divergence, and (ii) the overlap ratio for the chaining algorithm even though, as shown in [35], OC results are highly robust with respect to this parameter. MGA, MAUVE, and ProgressiveMAUVE include additional parameters linked to the four phases strategy, for instance the lengths of the matches that are used in the first and the third phases (P1 and P3) are critical. Moreover, the parameters of MAUVE/ProgressiveMAUVE need to be adjusted to the level of nucleotide divergence among the genomes to be aligned, even at the intra-species level. Therefore MGA and MAUVE/ProgressiveMAUVE are difficult to incorporate in large-scale automated studies. The use of local alignments selected on their E-value makes YOC relatively independent of this problem. Evidence for this is the higher coverages achieved with YOC on more divergent species like *L. lactis.*
3. Simpler genome alignment result: the dramatically lower number of alignment segments and, consequently, an increase in their size compared to concurrent aligners (see *L. lactis* case study). Indeed, it is not trivial to examine, to check, or to use an alignment split into a large number of segments.

These features make YOC simpler and easier to use and to parameterize.

In addition to YOC, we provide a large benchmark of several genome aligners and introduce an original criterion, GRA-FIL, to evaluate the quality of a genome alignment. The filtration procedure we developed makes it possible to obtain a high-quality alignment backbone as a result of the post-processing of the raw alignments.

Concerning limitations, YOC does not deal with complex rearrangements, e.g. translocations, is designed for pairwise alignment only, and lacks a user graphic interface to visualize the results. Not dealing with translocations limits its use to collinear genomes, thus mainly (but not restricted to) bacteria, on which we have focused in this paper. Indeed, although less numerous than bacterial genomes, collinear eukaryotic genomes (or collinear parts of genomes) can also be compared with YOC, as the size of the genomes is not a direct limitation of the method. Unfortunately, extending the framework to deal with rearrangements means moving to a NP-complete problem, which becomes even more complex when proportional overlaps between fragments are accepted. In this context, multiple alignment is yet another layer to add to the complexity of the task, which seems premature given that pairwise alignment is not yet completely solved. Regarding the lack of a graphic interface, several tools like ACT [43], Artemis [44], GBrowse [45] or MOSAIC [16], propose adaptable graphical viewers that can be used with YOC.

Finally, our study identified several difficulties in comparing WGA tools. Some criteria are indeed difficult to compare. For example, the number of aligned segments, a measure of the alignment fragmentation, is not directly comparable between MAUVE, ProgressiveMAUVE and the other genome aligners: for MUMmer (NUCmer), MAUVE and ProgressiveMAUVE it represents the number of Locally Collinear Blocks (LCBs) in the alignments, i.e. roughly the number of inversions and translocations; for MGA and YOC, it is the number of segments that are interrupted by insertions/deletions and local inversions (for YOC only). Consequently, we still need dedicated resources, like the Mosaic database [16], to incorporate and compare genome alignments according to unified criteria.

## Conclusion

YOC is an efficient and sensitive new alignment software, which is easy to use and fast. It produces reliable pairwise bacterial genome alignments using a simpler strategy than most existing tools.

## Additional files

Additional file 1:
**Full results for Dataset 1, including 174 pairs of collinear genomes (45 bacterial species) aligned with YOC, MGA, MAUVE and LAGAN.** Columns show accession numbers (Ident), length of the alignment in kb (Len), mean coverage in % (Cov%), mean percentage of identity (Id%), mean coverage in % after GRAPe filtering (CovF%), and mean percentage of identity after GRAPe filtering (IdF%) for YOC, MGA, MAUVE and LAGAN alignments. Additional file 2:
**Full results for Dataset 2, on the subset containing 14 pairs of genomes of the** ***Lactobacillus*** **genus, aligned with NUCMER, MGA, MAUVE, ProgressiveMAUVE and YOC.** Columns describe each genome (accession number, length, species/strain name) and all the indicators used: number of segments (Segments), length of the alignment (AlignLength, in number of positions), mean coverage (COV, in %), number of identities (Ident in number of nucleotides) and mean percentage of identity (mean %id) for NUCMER, MGA, MAUVE, ProgressiveMAUVE (PMAUVE) and YOC alignments before (raw) and after GRAPe filtering. Additional file 3:
**Full results for Dataset 2, on the subset containing 55 pairs of genomes of the** ***Bacillus cereus*** **species, aligned with NUCMER, MGA, MAUVE, ProgressiveMAUVE and YOC.** Columns describe each genome (accession number, length, species/strain name) and all the indicators used: number of segments (Segments), length of the alignment (AlignLength, in number of positions), mean coverage (COV, in %), number of identities (Ident in number of nucleotides) and mean percentage of identity (mean %id) for NUCMER, MGA, MAUVE, ProgressiveMAUVE (PMAUVE) and YOC alignments before (raw) and after GRAPe filtering. Additional file 4:
**Full results for Dataset 3, including 21 collinear pairs exhibiting increasing genomic divergence, aligned with YOC, MUMmer (NUCmer), MAUVE and ProgressiveMAUVE.** Columns describe each genome (species/strain name, accession number, length), MUMi distance values, mean coverage of the alignment, percentage of identities of the alignment (%ident), percentage of gaps in the alignment (%gapAlignt), percentage of mismatches in the alignment (%mismatch alignt) for MUMmer, MAUVE, ProgressiveMAUVE (PMAUVE) and YOC. Additional file 5:
**Comparison of YOC and MAGIC results on 12 bacterial genome pairs.** The dataset was extracted from [8] and includes ten genome pairs corresponding to Table three of [8] and two distantly related bacteria pairs (*Chlamydia* and *Bacillus*) obtained from Table six of [8]. Columns give the type of pairs, the species name, the number of aligned segments produced by YOC, the number of Reorder Free (RF) segments produced by MAGIC and coverage as a percentage of the genome length (computed on the fragments that are part of the chain for YOC, and on the RF segments for MAGIC).
